# Translation and adaption of the interRAI suite to local requirements in Belgian hospitals

**DOI:** 10.1186/1471-2318-12-53

**Published:** 2012-09-07

**Authors:** Nathalie IH Wellens, Johan Flamaing, Philip Moons, Mieke Deschodt, Steven Boonen, Koen Milisen

**Affiliations:** 1 Center for Health Services and Nursing Research, Kapucijnenvoer 35 – PB 7001/4, B-3000, Leuven, Belgium; 2 Leuven University Division of Geriatric Medicine, University Hospitals Leuven, Leuven, Belgium; 3 Leuven University Center for Metabolic Bone Diseases, Leuven, Belgium

**Keywords:** Aged, Geriatric assessment, Inpatient, interRAI Acute Care, Minimum Data Set, Validation studies, Instrument translation

## Abstract

**Background:**

The interRAI Suite contains comprehensive geriatric assessment tools designed for various healthcare settings. Although each instrument is developed for a particular population, together they form an integrated health evaluation system. The interRAI Acute Care Minimum Data Set (interRAI AC) is tailored for hospitalized older persons. Our aim in this study was to translate and adapt the interRAI AC to the Belgian hospital context, where it can be used together with the interRAI Home Care (HC) and the interRAI Long Term Care Facility (LTCF).

**Methods:**

A systematic, comprehensive, and rigorous 10-step approach was used to adapt the interRAI AC to local requirements. After linguistic translation by an official translator, five researchers assessed the translation for appropriate hospital jargon. Three researchers double-checked for translation accuracy and proposed additional items. A provisional version was converted into the three official languages of Belgium—Flemish, French, and German. Next, a multidisciplinary panel of nine experts judged item relevance to the Belgian care context and advised which country-specific items should be added. After these suggestions were incorporated into the interRAI AC, hospital staff from nine Flemish hospitals field-tested the tool in their practice. After evaluating field-test results, we compared the interRAI AC with Belgian versions of the interRAI HC and interRAI LTCF. Next, the Flemish, French, and German versions of the Belgian interRAI portfolio were harmonized. Finally, we submitted the Belgian interRAI AC to the interRAI organization for ratification.

**Results:**

Eighteen administrative items of the interRAI AC were adapted to the Belgian healthcare context (e.g., usual residence, formal community services prior to admission). Fourteen items assessing the ‘informal caregiver’, and 17 items, including country-specific items, were added (e.g., advanced directive for euthanasia).

**Conclusions:**

The interRAI AC was adapted to local requirements using a meticulous and recursive 10-step approach. As use of the interRAI Suite continues to grow worldwide and as it continues to expand to other care settings and populations, this procedure can guide future translations. This procedure might also be used by others facing similar challenges of complex translation and adaptation situations, where multidimensional instruments are used across multiple care settings in multiple languages.

## Background

Older persons receive multiple care services and are treated at different care facilities. In light of continuity of care, the need for uniform communication and good-quality data transfer systems is growing. This need is met by the interRAI system. The interRAI Suite includes comprehensive assessment instruments designed for a range of clinical services across multiple care settings (e.g., interRAI Mental Health, interRAI Palliative Care) [1,2]. It is the first assessment method that facilitates data transfer between caregivers based on a common set of standardized assessment items [3,4]. Although individual interRAI instruments are used in more than 30 countries, Belgium is one of the first countries to test the connection between various instruments in clinical practice. In this regard, an internet-based system has been developed to follow geriatric patients’ treatment across multiple care settings and to track patient functioning longitudinally. Continuity of care becomes increasingly important due to shorter hospital stays and early discharge planning [5]. Standardization lays the foundation of a common language, improves efficient communication, and implies evidence-based clinical outcome measures, quality indicators, and benchmarking [3].

InterRAI is a not-for-profit network comprising researchers from over 30 countries; their aim is to improve care for disabled patients and those with complex disorders [1]. In 1980, it began developing the set of assessment instruments comprising the interRAI Suite and since then has extensively tested and refined the suite for use in practice. The interRAI Acute Care Minimum Data Set (interRAI AC) was released in 2006. It is tailored to assess frail hospitalized older persons [6] and is a vital link for data transfer between different healthcare services. When implementing the interRAI AC in a country, one should take into consideration cultural, social, and healthcare contexts. Therefore adaptation to the local requirements is needed. However, its content has been defined and refined based on validity studies by interRAI [2,6] and cannot be changed unconditionally. Although the interRAI AC is available in six languages [1], no studies have reported how it has been translated. A search using the terms “Resident assessment instrument” or “interRAI” or “Minimum Data Set”, AND “translations”[Mesh] yielded only one article in PubMED. This article only briefly addressed the translation and adaptation process of the interRAI Long Term Care Facility (LTCF) instrument, which was designed for residential care [7].

Methodological difficulties exist in translation processes that could compromise the validity of the interRAI Suite [8]. The challenge is to adapt each version of the instrument to the relevant context and to a form that is comprehensible, while maintaining the meaning and intent of the original items [9,10]. Failure to achieve this end point might lead to the conclusion that different geriatric profiles exist, when in fact the items may have been interpreted differently by the clinicians because of linguistic shortcomings and unsatisfactory modifications [9]. It also may interfere with the reliability and validity of patient outcomes on the assessed geriatric domains. General methods have been developed to minimize this problem, but few guidelines exist and no consensus is available [8,10-13].

This report is the first to deliver an adaptation method that takes into account the complexity of the interRAI Suite and that tackles the challenges of the multidimensional character of the individual instruments, while preserving both the individuality of the multiple setting versions as the link between them. Our aim was to translate and adapt the interRAI AC instrument and its manual for use in Flemish hospitals. We used a systematic process that took into account the complex context in which the three interRAI instruments (interRAI Home Care (HC), interRAI LTCF, interRAI AC) are used jointly in one BelRAI web application. We aimed to transfer patient data across three care settings in a country with three official languages (Flemish, French, German).

## Methods and results

### Instrument

The interRAI AC (version 9, 2006) consists of 98 standardized clinical items that sample 12 domains, including patient history, cognition, communication, mood and behavior, activities of daily living, continence, nutrition, pressure ulcer, medical diagnosis, health condition, medications, and discharge potential. All items and their scoring options are explained in detail in an extensive manual. Furthermore, the interRAI AC is designed to map fluctuations in a patient’s functioning over time and across four assessment periods (premorbid, admission, reassessment, and discharge) [6]. The premorbid assessment links prehospitalization and admission data and serves as a baseline reference for the patient’s capacity in the rehabilitation process.

**Figure 1:**
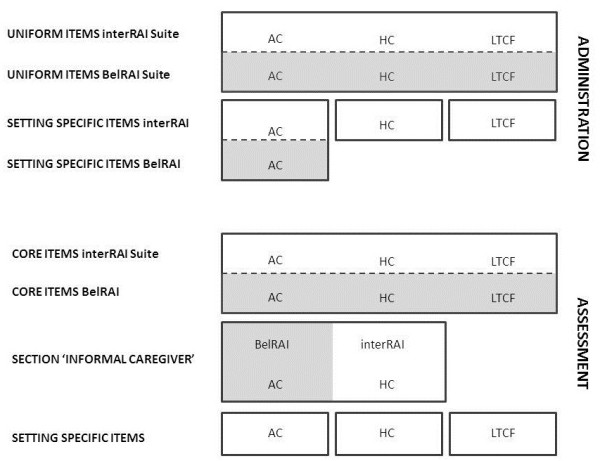
**Architecture of the interRAI Acute Care instrument and its adaptation to the Belgian care context.** BelRAI, The Belgian portfolio containing interRAI instruments adapted to the Belgian care context; AC, interRAI Acute Care instrument; HC, interRAI Home Care instrument; LTCF, interRAI Long Term Care Facilities. Boxes marked in grey indicate the parts of the interRAI AC instrument that are adapted to the Belgian hospital context or to the Belgian interRAI Suite, which includes the interRAI HC and the interRAI LTCF.

There are three types of items (Figure 1). Firstly, there are *core assessment items* of the interRAI Suite. These items evaluate universal geriatric syndromes. Therefore these core items appear in various instruments of the interRAI portfolio. Since interRAI introduced third-generation comprehensive geriatric assessment, these core items are identical in order to enable unambiguous communication across settings. Moreover, these standardized items are defined, scored, and phrased in the same way, as they refer to exactly the same construct in all settings [1]. Secondly, there are *setting-specific assessment items* that are relevant in a specific setting and cannot be found in any of the other instruments of the interRAI Suite. Thirdly, next to the assessment items (e.g. type 1 and 2), the instrument contains *administrative items*.

With regard to the translation and adaptation process, different strategies should be applied according to the item type. In translating and adapting the core items, one should ensure that the wording of an item matches exactly across all instruments. For example, in Belgium, the standardized assessment is implemented simultaneously in three settings. Thus, the items assessing delirium should be identical in the interRAI HC, the interRAI LTCF, and the interRAI AC. Furthermore, the items that appear solely in the interRAI AC should fit the Belgian hospital context. Lastly, administrative items need extra attention and need to be studied rigorously, because content and relevance may differ greatly across countries. Certain administrative items may be nonexistent (e.g., indigenous status in the Australian version) and/or very rare (e.g., hospital insurance status). In addition, the scoring categories need to be redefined (e.g., types of residence).

### Ten steps to translate and adapt the interRAI Suite

**Figure 2:**
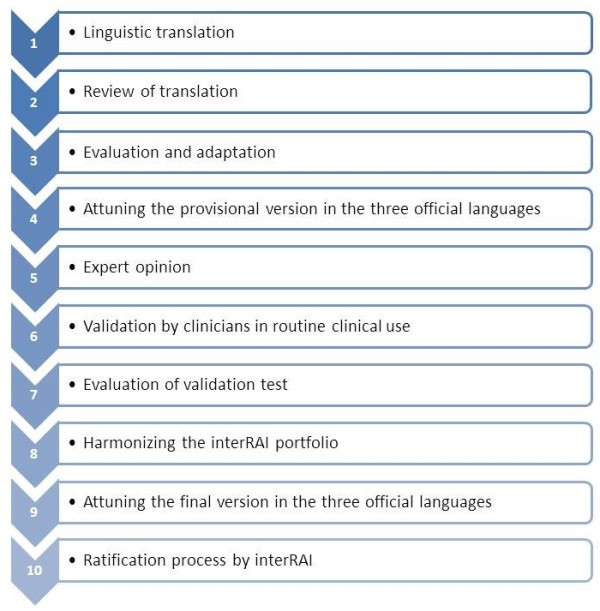
Ten steps for translating and adapting the interRAI Suite.

To translate and adapt the interRAI AC, we performed a rigorous step-by-step recursive process (Figure 2) based on the general guidelines of Geisinger [12]. As he suggested, we designed the procedure according to the specific needs of the current topic, language, and setting. For some phases, the guidelines of Guillemin et al. were used [14]. A similar process was conducted in the three language regions of Belgium to ensure that the final results would be uniform. The main focus of the current paper is the Flemish adaptation process. However, as Belgium has three official languages, the translation and adaptation process in one language is inevitably related to the translation process in the other languages. The multiple-step method we applied might be useful for other countries that face a similar challenge with multiple languages.

#### Step 1: Linguistic translation

First, the original and official English version of the interRAI AC and its manual were translated into Flemish by a qualified translator on an item-by-item basis [14].

#### Step 2: Review of translation

Conversion of the original English version to a Belgian version entails more than a purely linguistic translation. The content of the translated items must correspond idiomatically to the geriatric terminology used in Flemish acute hospitals [10]. To overcome the translator’s problem that he may not be sufficiently knowledgeable in the specific area of comprehensive geriatric assessment (CGA) [9], a committee of five researchers with (geriatric) nursing or paramedical background independently examined the translated version. The researchers were part of the Belgian Acurate.be research group. All were bilingual, had good knowledge of English, and were familiar with interRAI and local hospital context. They carefully and systematically reviewed the quality of the translation item-by-item, comparing the original and the translated versions. One of the researchers, ‘the instrument adapter’, compiled all remarks and suggestions about geriatric jargon.

#### Step 3: Evaluation and adaptation

The translated version, along with all suggestions of the previous phase, was then independently evaluated by three members of the coordinating group, which comprised one geriatrician and two advanced practice nurses in geriatrics. They double-checked *the accuracy of the translation* and checked the extent to which the translation appropriately fit the acute care context. *All jargon-related suggestions* were evaluated, and each reviewer separately selected the wording that best fit the Flemish hospital setting. Furthermore, they examined whether the assessment instrument had any oversights or omissions with respect to content, paying special attention to the administrative sections. Adaptations of the assessment content and the administrative items were proposed. Again, the instrument adapter collected all suggestions. After sharing the comments with one another, the group met to consider the points made by each of the eight reviewers (of steps 2 and 3) in order to reconcile any differences in opinion, a process proposed by Geisinger [12]. During this process, the adapted version was formally and iteratively compared with the original source-language version. This process enabled us to identify potential problematic items and to reassess and retranslate them until we were confident that the items would be interpreted in the same manner in both languages. All these changes resulted in a provisional revised translation and adaptation. In this phase, an intermediate version of the manual was adapted as well.

The results of steps 1 to 3 were organized on three levels: instrument translation and linguistic fine-tuning, adaptation of the administrative sections to the local care context, and suggestion of additional assessment items conform with CGA used in routine clinical practice in Belgian hospitals. More detailed information is found in Appendix 1.

#### Step 4: Standardizing the provisional version of the interRAI AC across the three official Belgian languages

The content of the provisional versions of the Belgian interRAI AC in Flemish, French, and German was standardized in order to achieve identical content. Three instrument adaptors native to the three Belgian language regions discussed the content of the administrative items until consensus was reached.

#### Step 5: Expert opinion

The test version plus a questionnaire was sent by e-mail to partners of the Acurate.be research group. A multidisciplinary expert panel [14] of nine Flemish-speaking clinicians participated: two geriatricians, one general practitioner, four case managers, one nurse, and one interRAI member. They were all familiar with CGA and had no prior knowledge of the interRAI AC [15]. These experts all volunteered, were independent of the researchers, and did not include the translator. Their task was threefold: (1) identify parts differing from the original, (2) examine item relevance, (3) assess readability and accuracy. Their main purpose was to identify parts of the test version that significantly differed from the original instrument. These were the administrative sections and the items added by the reviewers that were, according to their opinion, indispensable in geriatric assessment in the Belgian context. For this purpose, the experts were asked to evaluate on a four-point scale the relevance of the items and scoring categories of the selected parts (i.e., the sections ‘identification information’, ‘intake and initial history’, ‘assessment dates’, ‘discharge potential’; plus additional Belgian items and the additional section ‘informal caregiver’), as suggested by Lynn [16]. Analyses of content validity index (CVI) and modified kappa were conducted according to Polit and Beck [17]. The methodology followed that of a previous study and is described in detail elsewhere [18]. In addition to item relevance, the experts evaluated readability, level of ambiguity, completeness, and accuracy. The results of this phase are reported in detail in Appendix 1.

The revision was approved by the research group, and when necessary, problematic items or scoring categories were discussed once more. This resulted in a version ready for field-testing with identical content in the three languages. The manual was adapted accordingly.

#### Step 6: Validation by clinicians in routine clinical use

Several methods can be used to validate the translation. According to Sperber, none is fail-safe [10]. Within this study, the clinical relevance of each item was evaluated in nine geriatric and eight non-geriatric acute wards of nine Flemish hospitals. Item-level relevance was systematically evaluated according to clinicians’ opinions [14]. Frequency distribution, missing values, and invalid scoring of the interRAI AC were recorded and evaluated in 256 geriatric patients admitted to hospital [15]. The methodology and results are reported in detail elsewhere [18]. After testing in clinical contexts, the next stage in the adaptation process was to identify problematic items and to judiciously decide which revisions were needed.

#### Step 7: Evaluation of the validation test

On the basis of all collected information, the three test adaptors of each language region independently revised the tested versions. Dissenting suggestions were evaluated and discussed by the Acurate.be research group until consensus was reached. The researchers kept in mind all previous suggestions and ensured the instrument was consistent with the original instrument [14]. Based on the findings of the previous phase, some structural changes were made. The original manual prescribes assessment for four periods: premorbid, admission, day 14, and discharge. In clinical practice, the ‘day 14’ period is too rigid. In some cases, reassessment can be unnecessary, even though hospitalization exceeds 14 days. In some cases, reassessment is desirable prior to the 14^th^ day after admission. For this reason, the ‘day 14’ assessment was replaced with the more general phrase ‘reassessment’. Thus, when to reassess a patient is left up to the clinicians. Users are instructed during training and in the manual that, after a period of approximately 14 days, a new assessment is advisable. Furthermore, in the original interRAI AC, some assessment items are excluded for specific assessment periods (e.g., no premorbid assessment of delirium symptoms). According to the clinicians’ opinion, some excluded items should be used for all assessment periods, because systematic monitoring seems important during hospitalization (e.g., easily distracted, episodes of disorganized speech, mental functioning varies over the course of the day, acute change in mental status from baseline, mode of nutritional intake, fatigue, most severe pressure ulcer). Furthermore, in addition to listing community services prior to admission, these services should also be listed at discharge.

Next to the structural changes, clinicians provided suggestions about adding, removing, and adjusting assessment items. Details are listed in appendix 8.

#### Step 8: Harmonizing the interRAI portfolio

Since the interRAI AC would not be used as a stand-alone tool, but would serve as a link in data transfer between settings, we compared the phrasing of all common items and scoring options in the Belgian versions of the interRAI AC, interRAI HC, and interRAI LTCF. The aim was to agree on the content of the administrative sections and the core assessment items in order to link the three instruments perfectly. This uniformity should allow dependable data transfer across care settings. Within each language region, a consensus meeting was organized with the instrument adapters. Subsequently, a committee of two Flemish, two Wallonian (French-speaking), and one German-speaking researchers discussed the final problematic items.

We strived for balance between optimal wording and respecting the universal character of an item. For 65 items, the wording of the item description or the scoring options differed between the interRAI AC, the interRAI HC, and interRAI LTCF. Although these differences might have been very small, they were all listed and were discussed item-by-item. To optimize readability and fluency, the best phrasing was chosen. This means that in some cases the phrasing in interRAI HC and interRAI LTCF was adjusted according to the results of the current adaptation process. In some other cases, the wording of the interRAI HC and interRAI LTCF was chosen. Results are described in Appendix 1.

#### Step 9: Standardizing the final version of the interRAI AC across the three official Belgian languages

The results of each language region were consolidated. We carefully compared the different versions of the interRAI AC in the three official languages to ensure that each item and scoring option matched. This step enabled us to identify potentially problematic items and re-translate them until we were confident that the items would be interpreted in the same manner in all languages (i.e., Flemish, French, and German). In case the content differed, some adjustments were made. Furthermore, we added ‘mobile phone number’ of the general practitioner and removed ‘e-mail’. For the scoring options of ‘route of drug administration’, ‘vaginal’ and ‘auricular’ were added. Once again, the interRAI AC versions in the different languages were compared item-by-item to the other Belgian instruments. The committee judged that the instruments were equivalent in the three languages.

Throughout the process clinicians and researchers faced situations in which words were interpreted differently. Based on their reports, we tried to remove all ambiguous phrases. The difficulties experienced by the clinicians and researchers were noted in the instrument’s manual. In order to minimize ambiguity, additional information, phrases, and examples were added where needed to inform the users on how to administer, use, and interpret scores.

#### Step 10: Ratification by interRAI

Throughout the process, the interRAI regulations for adaptation were respected. Although the regulations allow items to be adjusted, the adjustments may not exceed more than 15% of the total item content. Adding country-specific items is allowed. However, the scoring options of the assessment are fixed. The scoring options for the administrative items can be changed, but recoding them into the interRAI categories should be possible. In July 2008, the interRAI AC was sent to interRAI to start the ratification process, which is regulated by interRAI. Seventeen items were added to the original assessment content and an additional section dealing with informal caregivers, which included 14 items, was added. Eighteen items were adjusted; two items were removed. Some structural changes for the assessment periods were made. Details are listed in Table 1. All adaptations were according to interRAI regulations. After ratification, the copyright remains with interRAI. However, interRAI generally grants governments, service providers, and researchers royalty-free licenses in exchange for data that are collected for clinical use or research purposes [1].

**Table 1:** Outline of the adjustments in the Flemish interRAI AC

**ADJUSTED**	**REMOVED**	**ADDED**
- Birth date	- Name: middle initial	- Admitted from & usual residence
- Marital status	- Current payment sources for inpatient stay	- Wishes or needs related to nourishment or personal hygiene, etc.
- Ethnicity/race		- Contact persons – general practitioner: address, telephone & mobile number
- Primary language		- Contact person: address, relation to patient, telephone and mobile number
- National numeric identifier		- Hearing appliance
- Facility/agency provider number		- Visual appliance
- Date stay began		- Weight at discharge
- Admitted from		- Prescription diet
- Living arrangement		- Treatments: others
- Time spent in emergency room		- Therapy/nursing: nursing services
- Date of surgical procedure		- Therapy/nursing: others
- Reference date of admission assessment		- Advance directive for euthanasia
- Reference date of day 14 assessment		- Advance directive: other
- Reference date of discharge assessment		- Community services: night care
- Primary mode of locomotion: bedbound or confined to chair		- Community services: personal alarm system
- Treatments: intravenous therapy		- Community services: physiotherapy
- Discharge: last day of stay		- Community services: pedicure
- Discharge: discharged to		- Additional section: social support/informal care giver (14 items)
**STRUCTURAL CHANGES**		
1.	** "Day 14" assessment period replaced with "Reassessment"**	
2.	** Reassessment of following items:**	
	Easily distracted	
	Episodes of disorganized speech	
	Mental functioning varies over the course of the day	
	Acute change in mental status from person’s baseline	
	Mode of nutritional intake	
	Fatigue	
	Most severe pressure ulcer	
3.	** Discharge assessment of following items:**	
	Community services	

## Discussion

The aim of this study was to translate and adapt the interRAI AC to the Flemish hospital setting and Belgian care context in compliance with interRAI regulations. We used a combination of translation techniques [8,11,12] to address the complex nature of the interRAI Suite, which was to be applied in the complex Belgian context. This context is complex because three interRAI instruments of three care settings are used conjointly in one web application and because Belgium has three official languages. Therefore, the process of adapting the Flemish interRAI AC could not be done in isolation. We had to approach the adaptation process, appreciating its particular background/perspective and taking into account the implications of adaptation at multiple levels.

*On a hospital level*, the assessment file needs to contain all the items that are usually assessed by routine CGA in Belgian geriatric wards. For this reason, we involved clinicians of multiple disciplines (e.g., nurse, geriatrician, case manager). Both academic clinicians and non-academic clinicians reviewed the administrative sections and indicated how these sections should be adjusted to the Belgian care setting. Furthermore, they suggested which topics (e.g., informal caregiver) should be added in order to be comparable with routine acute assessments (steps 3, 5, 6).

*On an assessor level,* the wording needs to fit the jargon currently used in Flemish geriatric literature and in clinical practice. The phrasing must be fluent and unambiguous to the assessors in clinical practice [14] (steps 2, 3, 5, 6). The scoring of clinical situations sometimes requires explanations or examples that cannot be fully documented in the interRAI AC itself. Therefore, the manual should complement the instrument. Within the Belgian software (BelRAI), this additional information is near at hand, as the assessment items are all linked to specific pages of the manual.

*On a patient level*, various caregivers will consult interRAI assessments carried out in hospitals, as patients move across several care facilities. In order to track a patient’s health and functional status longitudinally, the adaptation procedures had to guarantee that the core set of items in the interRAI HC, interRAI LTCF, and interRAI AC instruments remained identical, both formal and semantically (step 8).

The interRAI AC is part of the Belgian interRAI (BelRAI) portfolio, which will be implemented in three language regions: Flanders, Wallonia, and the German community. *On a national level*, the aim was to centralize patient data. Therefore we recursively evaluated interim versions to harmonize the draft and final versions across the three official languages throughout the adaptation process (steps 4, 9). In addition to centralization, harmonization across languages is beneficial for using the BelRAI portfolio in bilingual regions, so colleagues within the same hospital can complete a shared assessment in the preferred language. Furthermore, if a patient moves to another region or if a caregiver speaks another language, previous records can be consulted in the language of choice.

*On an international level*, there is a need for reliable, large datasets for cross-national comparison of geriatric care in order to increase geriatric knowledge. Therefore, the process of adapting the Flemish interRAI AC instrument was done rigorously. During the adaptation procedure, the official source instrument served as a reference and was consulted repeatedly and systematically every time an item was adapted (steps 1 to 9) [14].

*On an interRAI level*, the interest of different nations in using the interRAI Suite continues to grow. It is of utmost importance that the initial content is preserved. There are regulations for permitted adaptations; interRAI retains the copyright to the instrument. We followed the interRAI regulations and submitted the Belgian portfolio for careful examination and official approval (step 10).

The application of this systematic and iterative 10-step approach (Figure 2) produced the Flemish version of the interRAI AC. We are confident that the adapted instrument closely resembles the content in the standard version. This conclusion, however, must be qualified, with the understanding that it is impossible to achieve 100% validation [9]. Also, one can always argue that significant differences in cross-national use could be the result of methodological flaws rather than actual differences [9]. We believe that the careful step-by-step process of validation described in the present study reduces the latter possibility to an acceptable minimum. However, the procedure described in this paper is only a first step of a larger process, involving extensive psychometric research aimed at obtaining a wide and diverse body of evidence about various aspects of validity [19], reliability [20], and responsiveness.

Thus far, psychometric evidence on the original version of the interRAI AC is scarce and is limited to draft versions [2,6,21]. The results of the current research must be interpreted within this context. Furthermore, this process resulted in a first Flemish version of the interRAI AC. Belgium is the first country to test and use multiple instruments of the interRAI portfolio simultaneously in transitional care. The wording of some specific items was different across the interRAI HC, interRAI LTCF, and interRAI AC instruments (e.g., nausea versus vomiting). Our approach in comparing these instruments in a meticulous process revealed these differences. More research is needed to harmonize all instruments of the interRAI portfolio. InterRAI considers the development of these instruments to be dynamic: These instruments can be optimized and revised in upcoming years as more clinical experience is gained [1].

At this stage, we noticed that the desired adjustments did not always match the possible adjustments. In other words, the suggestions made by experts and clinicians on how the interRAI AC instrument would best fit the acute context could not always be put into practice. There were constraints. Uniformity with the InterRAI HC and InterRAI LTCF was a priority, because small differences in wording or scoring would imply problems in the reliability of transmural data transfer. Also, since no overall scores are calculated in the interRAI method, the items are regrouped into clinical assessment protocols (CAPs) and scales defined by interRAI. Altering items would affect the clinical algorithms of the output. Moreover, some adjustments are unavoidable in the perspective of instrument integration, even if the clinicians did not mention these. For example, the word ‘patient’, which is common in the acute care sector, was changed to ‘client’ due to practical reasons having to do with the BelRAI software architecture. Another example is intake data, with a more administrative character, which need to be uniform across the interRAI portfolio.

There is no gold standard for translation techniques [8,10-12]. Rather than performing a back-translation, we used multiple expert panels of differing constitution for pre-pilot evaluation and subsequent field-testing to carefully control the quality of the translation. According to Geisinger [12] and Cha et al. [11], this technique is more effective for ensuring that the translation and adaptation is conducted appropriately [12]. During each step, problematic items were identified. But before adjusting the instrument, the items were compared with their original counterparts and, when necessary, revised by the instrument adapter or a committee. Independent back-translation could be used in future studies to further validate the interRAI AC in the Belgian acute care context. Although the current translation and adaptation process was time-consuming, all the different steps were necessary. Because the goal was not merely to guarantee that items on the interRAI AC tap into the same construct but also to have confidence that each item and each scoring option across the instruments tap into the same construct. This procedure (Figure 2) might be used by others facing similar challenges of complex translation and adaptation situations in which multidimensional instruments will be used across multiple languages in multiple care settings. As the use of the interRAI Suite continues to grow worldwide and as the interRAI Suite expands to other care settings and populations, this procedure can guide future translations.

## Conclusions

Our aim was to translate and adapt the interRAI AC using a meticulous and recursive 10-step approach. Linguistic translation, review, and pilot testing were done in an iterative process in order to adapt the translation to geriatric jargon in the Belgian care context, to all three official languages in Belgium, and to the Belgian interRAI portfolio. Translation, review, and pilot testing were performed by a certified translator, experts, and clinicians, respectively. We carefully ensured that the core items appearing in the interRAI HC, interRAI LTCF, interRAI AC remained uniform. Although some adjustments were made to fit the Belgian context, the instrument was not altered in any fundamental way.

## Appendix 1. More detailed information regarding the results of the translation and adaptation process

### Steps 2 and 3: Review of linguistic translation, evaluation, and adaptation

In steps 2 (review of linguistic translation) and 3 (evaluation and adaptation), the translation was adjusted to *the acute care jargon* for 33 items. For example, for the items concerning delirium, pressure ulcer, urinary collection device, (instrumental) activities of daily living, nutritional intake, treatments, etc., the literal translation did not match the wording of well-known expressions used in Flemish literature and clinical practice. Furthermore, if necessary, item *wording and phrases were fine-tuned* to be more readable and to minimize vagueness or misinterpretation. In total, 37 phrases were rephrased. Examples are not provided because they are language specific.For *the administrative items*, content cannot be changed fundamentally. However, the Belgian care context is significantly different from the Australian context, where the original interRAI AC originates. So we aimed to *adjust* the administrative items as close to the local context as possible. For example, the scoring options of residential status (‘Where is the patient admitted from?’) were adjusted, because some of the options do not exist or are not relevant in Belgium (e.g., ‘board and care’). Thus, the discharge information was changed accordingly. The scoring categories of the country-specific items ‘primary languages’ and ‘ethnicity’ were replaced with the three official languages of Belgium and the major ethnic groups in Belgium, respectively. The order of the digits of all seven dates was changed (e.g., year-month-day was replaced with day-month-year). In total, 15 administrative items were adapted. Furthermore, three items were *added* (e.g. ‘religious conviction’, ‘the address and telephone number’ of the general practitioner and the contact person, and ‘usual residence’ was superadded to ‘admitted from’). Lastly, the item ‘current payment sources for inpatient stay’ was *removed*.At the end of the instrument, a section assessing *the informal caregiver’s situation* was *added*. Since the end of the 1990s, case management has been implemented nationwide in Belgian hospitals as a standard procedure for patients admitted to a geriatric unit [22]. Within this context, the assessment of social support and informal helpers is indispensable. We added 16 items from the interRAI HC and the interRAI Screener.

### Step 5: Expert opinion

Firstly, item relevance was judged. Overall, the average CVI-total was 0.91, indicating that the assessed administrative and informal caregiver items and their scoring categories met the criteria of excellent content validity (>0.90) [17]. However, the subscores revealed an average CVI of 0.94 and 0.84 for the administrative and the informal caregiver sections, respectively. Looking at the scores on an item level, the modified kappa was excellent for 59 administrative items and scoring categories (κ* ≥ 0.75), good for nine items (0.60 ≤ κ* ≤ 0.74), and poor for two items (e.g., name initials, nickname; κ* < 0.40). For the informal caregiver section, 24 items or scoring options were evaluated as having excellent relevance, and five as having poor relevance (e.g., ‘lives with person longer than six months’, ‘lives with person six months or less’, hours of informal care and active monitoring: ‘total time 5 weekdays’, ‘total time 2 weekend days’, ‘strong and supportive relationship with family’). Removing items having poor relevance would increase the average CVI to 0.96 and 0.91 for the administrative and informal caregiver sections, respectively, resulting in an increased CVI-total of 0.95. In preparation for the next phase, the items with poor relevance that also appeared in either the interRAI HC or interRAI LTCF instruments were identified but not removed yet in order to guarantee that the instruments remained as identical as possible.Secondly, the experts were asked to read through the assessment items and to check fluency and unambiguity. Five typographic corrections were reported. Three items were rephrased to decrease ambiguity (e.g., time since last hospital stay: scoring option ‘now in hospital’ was replaced with ‘transfer from other hospital’; living arrangement: ‘alone with spouse partner’ was replaced with ‘with spouse/partner only’; negation was removed in ‘informal helper is unable to continue in caring activity’). Textual changes were made in three items to improve readability.Thirdly, omissions with respect to content and corrections were reported. The administrative sections were revised to the Belgian care context. Omissions were reported for six items (e.g., community services: addition of ‘night care’ and ‘personal alarm system’; ‘email’ of the physical practitioner; ‘relation to the patient’ of the contact person; ‘residential status’: addition of two specific types of Belgian care institutions). These items were added for further relevance testing by clinicians during routine care in the next phase. Furthermore, remarks that improved the readability were implemented.

### Step 7: Evaluation of the validation test

Although the instrument was already long, the clinicians suggested adding 31 more items. On the other hand, they also wanted to simplify the scoring of 25 items by reducing or merging the scoring options. For 4 of the 25 items, they even preferred an open text field to standardized scoring options. In this evaluation phase, specific attention was paid to the core items that are mutual in interRAI HC, interRAI LTCF, and interRAI AC. Because of the standardized character of the interRAI portfolio, none of the suggestions for reducing the scoring options was granted. Only 5 of the 31 suggestions for additions were put into practice, because these items were also present in either the interRAI HC or interRAI LTCF (e.g., ‘respite care’ for residence status and discharge destination, ‘prescription diet’, ‘use of visual appliance’, ‘use of hearing appliance’, and ‘weight’ at discharge).

### Step 8: Harmonizing the interRAI portfolio

In line with the interRAI HC and interRAI LTCF, one item was added to the interRAI AC (‘advance directive for euthanasia’). Furthermore, religious conviction was replaced with a blank space, ‘wishes/special needs relating to food, personal hygiene, etc’. The scoring option ‘bedbound’ was rephrased to ‘bedbound or confined to chair’ for the item ‘primary mode of locomotion’. For drug frequency administration, ‘continuously’ was added. For ‘treatments’, IV medication was replaced with IV therapy, and the possibility to specify ‘other treatments’ was added. For ‘therapy/nursing services’, the item ‘respiratory therapy’ was removed and ‘nursing services’ was added. Here also the possibility to specify ‘others’ was added. ‘Physiotherapy’ and ‘patient sit-in’ were added to ‘community services prior to admission’. Lastly, throughout the instrument, the word ‘patient’ was replaced with ‘client’ to match the terminology in the interRAI HC and interRAI LTCF. We noticed that one item in the original English versions differed across the three instruments: In the interRAI AC, the item ‘gastro-intestinal status’ assesses ‘nausea’, whereas in the interRAI HC and interRAI LTCF the item assesses ‘vomiting’.

## Abbreviations

AC: Acute Care; HC: Home Care; LTCF: Long Term Care Facility; CAP: Clinical Assessment Protocol; CVI: Content Validity Index.

## Competing interests

The authors have no conflicts of interest.

## Authors’ contributions

NIHW, JF, PM, MD, SB, KM contributed to the study design and writing of the manuscript. KM was responsible for the study concept and overall execution of the study. All authors have read and approved the final draft of the paper.

## Pre-publication history

The pre-publication history for this paper can be accessed here:

http://www.biomedcentral.com/1471-2318/12/53/prepub
